# Smartphone-Based Hearing Screening in Noisy Environments

**DOI:** 10.3390/s140610346

**Published:** 2014-06-12

**Authors:** Youngmin Na, Hyo Sung Joo, Hyejin Yang, Soojin Kang, Sung Hwa Hong, Jihwan Woo

**Affiliations:** 1 School of Electrical Engineering, Biomedical Engineering, University of Ulsan, Ulsan 680-749, Korea; E-Mails: nayoungmin39@gmail.com (Y.N.); mywngprud1@gmail.com (H.S.J.); hjyang1990@gmail.com (H.Y.); ksj8920@hanmail.net (S.K.); 2 Department of Otorhinolaryngology-Head and Neck Surgery, Samsung Medical Center, Sungkyunkwan University, Seoul 330-714, Korea; E-Mail: hongsh@skku.edu

**Keywords:** smartphone, ubiquitous healthcare, hearing, audiometer, application

## Abstract

It is important and recommended to detect hearing loss as soon as possible. If it is found early, proper treatment may help improve hearing and reduce the negative consequences of hearing loss. In this study, we developed smartphone-based hearing screening methods that can ubiquitously test hearing. However, environmental noise generally results in the loss of ear sensitivity, which causes a hearing threshold shift (HTS). To overcome this limitation in the hearing screening location, we developed a correction algorithm to reduce the HTS effect. A built-in microphone and headphone were calibrated to provide the standard units of measure. The HTSs in the presence of either white or babble noise were systematically investigated to determine the mean HTS as a function of noise level. When the hearing screening application runs, the smartphone automatically measures the environmental noise and provides the HTS value to correct the hearing threshold. A comparison to pure tone audiometry shows that this hearing screening method in the presence of noise could closely estimate the hearing threshold. We expect that the proposed ubiquitous hearing test method could be used as a simple hearing screening tool and could alert the user if they suffer from hearing loss.

## 1. Introduction

Hearing loss is highly prevalent worldwide. The World Health Organization estimated that there were 360 million persons affected by hearing loss in 2012. Hearing loss is associated with a variety of factors. First, hearing loss is highly related to aging. Approximately one-third of persons over 65 years old have impaired hearing. This type of hearing loss is quite gradual. The chronic conditions of hearing loss possibly result in depression, loss of self-confidence, social isolation, and cognitive decline [1,2]. However, hearing screening and the proper treatment of hearing aids for the elderly who are hearing impaired could significantly improve quality of life in many areas [2]. Thus, for adults who are 65 years old or older, it is important to be aware of hearing loss at an early stage. Second, hearing loss is also related to the over-use of personal audio-devices. Recently, many people use mobile phones or portable music players. This usage significantly increases the hearing threshold [3,4]. Typically, people who are exposed to high sound levels daily by using a headset are potentially at risk for hearing loss. If people are aware of the progress of hearing loss, an intervention, e.g., the listening level and the exposure to loud music, would be provided to prevent progressive hearing loss [4].

As many people such as smartphone users, portable music listeners, and older people are not aware of hearing loss at an initial stage, they need to periodically test their hearing. A hearing test in a clinic, called pure-tone audiometry (PTA), is performed using an audiometer system in a soundproof room. However, some people have difficulty testing their hearing in a clinic [5]. Thus, more convenient and easily accessible hearing testing is required. Nowadays, many people use a smartphone as well as a smart device. Because of their convenience, many mobile applications related to u-healthcare have been developed to monitor people's health [6–10]. The use of u-healthcare applications may help predict health conditions such as blood pressure and heart rate. Several research groups have studied hearing testing using a smartphone [11,12]. The hearing-test applications could be conveniently and alternatively used to check the hearing level instead of audiometry by a healthcare clinician.

As a smartphone application provides easy accessibility, people could perform a self-test anytime and anywhere in their daily life. Even though the test should be conducted in a quiet place, it is difficult to find a place as quiet as a soundproof booth. The smartphone users could run the application in a noisy environment such as a restaurant or subway terminal where the noise levels are generally between 40 to 70 dB SPL (sound pressure level). Thus, if the application test is conducted in noisy environment, the self-test system should compensate for the hearing threshold shift (HTS) resulting from background noise to estimate the real hearing level. However, no application includes such a compensation algorithm.

In this study, we have developed a hearing-test application that can be used at any place regardless of environmental noise. The application automatically corrects the noise-induced HTS by measuring the environmental noise using a built-in microphone in a smartphone. We have systematically analyzed the noise-induced HTS and developed an HTS correction algorithm on the basis of the recorded noise level. This paper describes the developmental procedure of the hearing-test application that includes the HTS correction and calibration procedure using a sound-pressure-level meter. Finally, we have validated the new application by comparison with the hearing level measured using a pure tone audiometer.

## 2. Materials and Methods

### 2.1. System Flowchart

**Figure 1.**
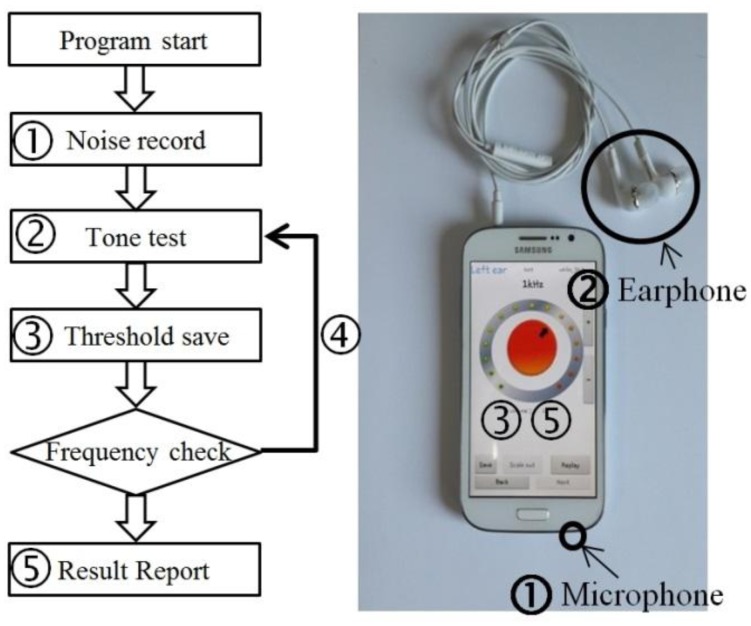
A flowchart of the application procedure showing the steps to test hearing in noisy environments.

Before explaining the methods of our study, we will briefly describe the screening procedure. Figure 1 shows a diagrammatic representation of an application process and a smartphone with an earphone. After running the application on a smartphone, a smartphone user selects the mode corresponding to the noise type that closely represents existing noise of the environment. There are two modes of either white noise or babble noise. As white noise contains all frequency components, it is frequently used to model a complex environment, e.g., a subway station, construction area, *etc*. The environmental sound in places such as a coffee shop or party room can be represented by babble noise. After the user chooses a noise mode, the smartphone automatically records the environmental noise over a period of 5 s using a built-in microphone (➀ in Figure 1). Then, the user plugs in earphones and begins pure-tone tests to estimate the hearing threshold of four frequencies: 500, 1000, 2000, and 4000 Hz (➁) [13].

During each pure-tone test, a 2 s duration tone is presented via the earphone. If the user could hear the frequency tone, the user adjusts the volume up or down by touching the buttons. By repeating this procedure, the user can save the level that is just starting to be heard by pressing the “save” button on the smartphone (➂), which is defined as hearing threshold. Once the “save” button is pressed, the “next” button for testing the next frequency is activated. Four frequencies are tested in the order of 1000, 500, 2000, 4000, and 1000 Hz, which is the sequence generally used in a clinic during pure-tone audiometry (➃). Because 1000 Hz is an important component in the audible-speech range, we repeated the 1000-Hz frequency test and averaged the two hearing thresholds [5]. After sequential testing from 500 Hz to 4000 Hz, the smartphone displays the hearing thresholds before and after correcting for the HTS that results from environmental noise (➄). The application also finally reports the hearing state as normal, mild, moderate, and moderately severe according to HL ISO 1964.

**Figure 2.**
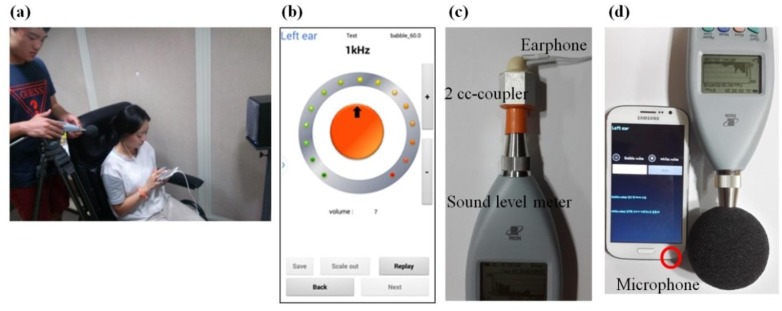
The application test in a soundproof room and the application GUIs for noise recording and the tone test.

Figure 2 shows an example of a scenario for developing and testing the hearing screening application (Figure 2a) and the captured GUI for the 1000-Hz hearing test (Figure 2b). All experiments for data acquisition were performed in a soundproof room to control for the background noise, and the subjects sat on a chair. Two loud speakers (Quad 9L Active, Quad Loudspeakers, Huntingdon, UK) were used to generate noise and were positioned at ±60° from the center line and 1 m apart from a subject.

### 2.2. Mobile Application Development

In this study, we have developed a mobile application based on an Android platform and tested the application using the Galaxy (Samsung, Suwon, Korea) smartphone. The application program was developed using Eclipse Kepler (Ver. 4.3) and the Android SDK 2.3.1 (API 9). The pure tones (500, 1000, 2000, and 4000 Hz, 2-s duration) were directly recorded from the clinic audiometer (Orbiter 922, GN Otometics, Taastrup, Denmark) using an analog-to-digital converter. Each tone has 0.03-s rising and falling times to enhance frequency specificity. We assumed that the environmental noise was either white noise or babble noise, which was composed using Matlab (Mathworks, Natick, MA, USA) and recorded at a busy street, respectively.

### 2.3. Calibration of the Earphone

To generate the test tones, we used an earphone packed in a smartphone package. The earphone delivers a tone sound for each frequency to test the hearing threshold. Before using the earphone, we calibrated the earphone by measuring the real output using a 2-cc coupler (HA-1, Etymotic Research, Elk Grove Village, IL, USA) and sound-level meter (Cesva, Barcelona, Spain) (Figure 2c). The sound output in a smartphone is generally controlled using a program unit from 0 to 15. Level 0 produces no sound, whereas level 15 produces the highest level sound. By calibrating the earphone, we could understand the real output level of the earphone in units of decibels SPL and not in program scales. Table 1 lists an example of the earphone calibration table for a typical 1000-Hz tone. The earphone units for controlling the volume output in a smartphone are listed in the left column. The real measured outputs in units of decibels SPL using a sound-level meter are listed in the center column. The sound level in units of decibels SPL was converted into a decibels-HL scale on the basis of ANSI S3.5-89, which are units for clinical use. For example, a 1,000-Hz tone at five on the program scale was generated via the earphone, and the real output was measured as 32.4 dB SPL (which is equivalent to 25.9 dB HL). After completing the earphone calibration table that describes the program units *versus* the real output, they were implemented into the application.

### 2.4. Calibration of the Microphone

**Figure 3.**
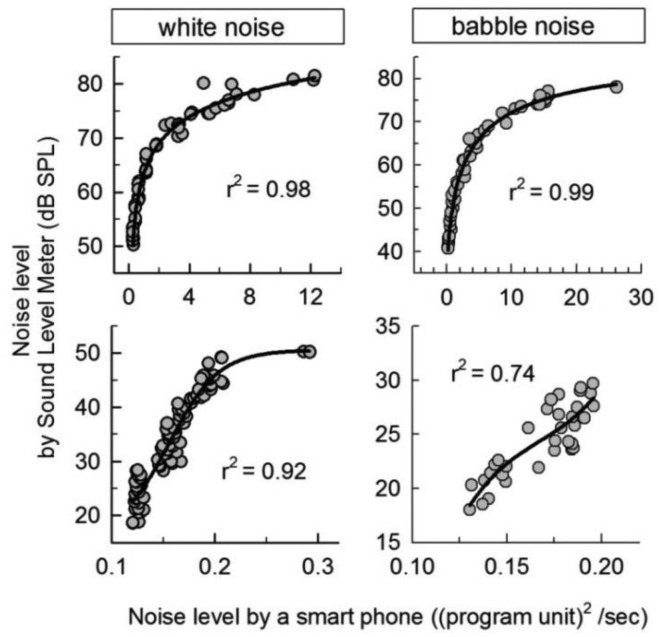
The noise-level relationships measured by a smartphone and a sound-level meter. White noise (left) and babble noise (right) were presented using two loud speakers. Circles represent data by repeated measures. Lines show the interpolated data using a discrete set of measured data.

To capture the environmental noise, a built-in smartphone microphone was used. Similar to the earphone calibration, the smartphone microphone was calibrated to provide a sound level in standard units (Figure 2d). The signal recorded by the smartphone microphone is represented in program units from 0 to 30 and not in decibels-SPL units. To complete a calibration table for the microphone, we calibrated the microphone using the sound-level meter and two loud speakers. Noise was generated using the loud speakers in a soundproof room. The smartphone and sound-level meter were placed 1 m apart from the speakers. Then, the sound was simultaneously measured using both the smartphone and the sound-level meter. Noise levels ranging from 0 to 80 dB SPL were calibrated to the system scale by decreasing the noise level in 5-dB steps using an attenuator (Texio, Yokohama, Japan). Figure 3 plots the noise level recorded by a smartphone *versus* the noise level recorded by the sound-level meter for white noise (left column) and babble noise (right column). The data set was fit to the equations listed on the analysis program and found to have a significant correlation coefficient (SigmaPlot, Systat Software Inc., San Jose, CA, USA).

For a more precise fitting, the data set was divided into two interval groups: 20–50 dB SPL and 50–80 dB SPL. The parameters of the fitting equations are summarized in Table 2. The variables “x” and “f” in Table 2 represent the average power ((program unit)^2^/s) measured by the smartphone microphone and the sound level (dB SPL) estimated as a real output value, respectively. Finally, the application could provide a physical sound level on the basis of this microphone calibration table when we measured the environmental sound using the built-in microphone.

### 2.5. Hearing Threshold Shift

The hearing thresholds for all frequencies (500, 1000, 2000, and 4000 Hz) were measured using the smartphone either in the presence of noise or in a quiet environment. The noise level was varied from 30 to 80 dB SPL in 10-dB steps, and the HTS was calculated by the difference in two hearing thresholds measured in the noisy and quiet environments. Thirty-six subjects who were 20–66 years old participated in this study. Fifteen subjects in the original group were tested to quantify the HTS. The human test in this study was reviewed and approved by the Samsung Medical Center Institute Review Board (Seoul, Korea).

### 2.6. Validation of the Application

Twenty-one subjects with normal hearing and three subjects with mild hearing loss (20–66 years old, 15 females and four males) participated in the validation of the application. They were tested with PTA and the smartphone in a soundproof room. When a subject tested their hearing with the smartphone, five different noise conditions (quiet, 40- and 60-dB-SPL babble noise, and 40- and 60-dB-SPL white noise) were presented using two loud speakers. To test the suitability of the application in real-world environments, one subject tested his hearing outside of a clinic on the street; at a café, bus stop, and public park; and inside of a bus. To assess the convenience of the application to the user, we surveyed the subjects with a questionnaire consisting of the following three questions: “Q1. How long have you used a smartphone: (1) never (2) 0–6 months (3) 6–12 months (4) 1–3 years (5) over 3 years. Q2. I think that the application is very complex to use: (1) strongly agree to (5) strongly disagree. Q3. I think that I need practice time of (1) less than 5 min (2) 5–15 min (3) 15–30 min (4) 30–60 min.”

## 3. Results

### 3.1. Effect of Noise on HTS

**Figure 4.**
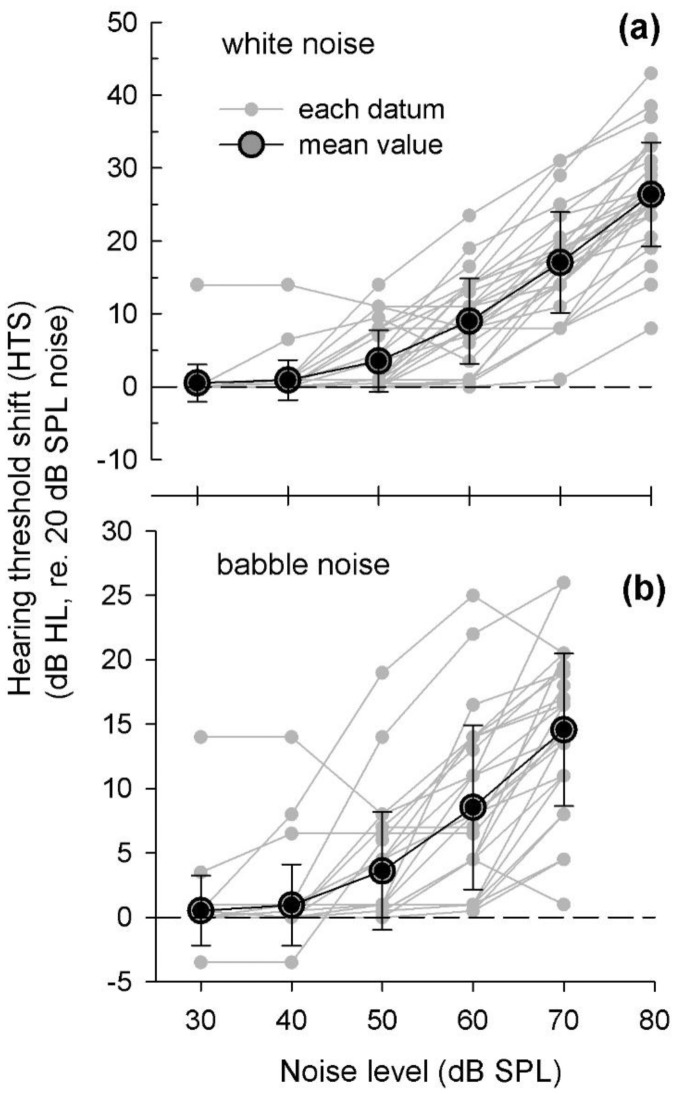
An example of the hearing threshold shift as a function of the white (**a**) and babble (**b**) noise level. Individual data (small grey circles, n = 15) and the mean hearing threshold shift are plotted *versus* the noise level.

Figure 4 shows an example of individual HTSs for a typical 1000-Hz pure tone *versus* the background noise level. Either white noise (a) or babble noise (b) was presented using two loud speakers. The individual HTS (small gray dots) and mean of individual HTSs (black circle) were plotted as a function of noise level. As the noise level increased, the HTS increases up to 45 dB HL. The HTS for white noise is higher than that for babble noise because white noise is continuously present and produces a greater masking effect than babble noise. To estimate the HTS at a typical noise level, linear regression equations at each interval (10 dB of noise level) were computed on the basis of the mean values.

**Figure 5.**
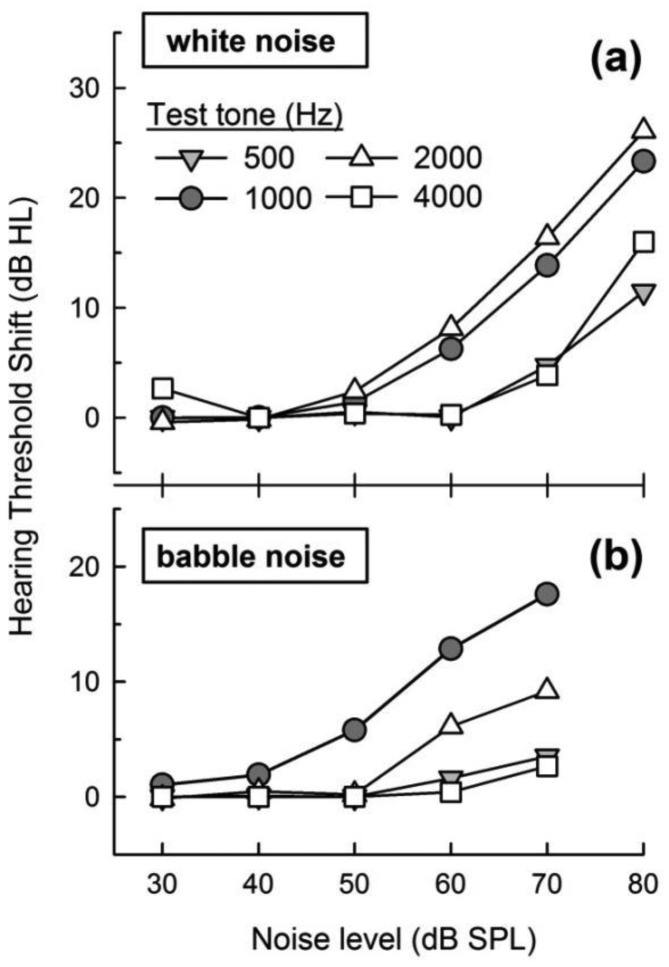
Mean of the hearing threshold shift for a tone (500, 1,000, 2,000, and 4,000 Hz) in the presence of environmental white (**a**) and babble (**b**) noise.

Figure 5 shows the HTS means of individual HTSs for four pure tones (500, 1000, 2000, and 4000 Hz) as a function of either the white-noise level (a) or babble-noise level (b). As seen in Figure 4, we calculated the HTS linear regression for each frequency data set. The linear regression between two known values could estimate the HTS for a typical noise level, e.g., the HTS at 55-dB-SPL noise is determined by a linear regression of the two HTS values at 50 and 60 dB SPL noise. Thus, once the application measures the environmental noise, the HTS was calculated and reflected to the correct hearing threshold.

Low environmental noise (below 40 dB SPL) did not affect the HTS, whereas noise greater than 50 dB SPL resulted in a greater HTS. A comparison of HTSs from the 500 to 4000 Hz tones indicates that the 1000 and 2000 Hz tones were more masked in the presence of environmental noise. The external noise has less of an effect on the 500 and 4000 Hz pure-tone tests, *i.e.*, the greater masking by noise in the 1000 and 2000 Hz interval is evident, presumably a result of the higher gain of the external ear.

### 3.2. Implementation of HTS on a Smartphone

We implemented the calibration tables for the earphone and microphone and the HTS tables into the application. When starting the application, the user selects the type of environmental noise. Then, the microphone measures environmental noise over a period of 5 s. HTSs for each frequency are calculated on the basis of the environmental noise level. Next, a user begins hearing testing using the earphone. A user might control the volume up and down until the minimum sound level of the pure tone below which a person is unable to detect the sound is achieved. After finishing the testing procedure for all frequencies, the application calculates the hearing threshold by correcting the HTS from the original measured threshold. Finally, the smartphone reports the hearing threshold before and after correction on the basis of the environmental noise level.

### 3.3. Comparison to the Standard Hearing Assessment

In order to evaluate the performance of the hearing-test application, we compared the hearing threshold measured by PTA and the smartphone without and with the HTS-correcting algorithm. PTA is a standard behavioral test widely used in a clinic. The hearing thresholds of three subjects were measured using PTA by an experienced audiologist, and the subjects also tested themselves using the smartphone. Before beginning the hearing test, the subjects practiced the use of the smartphone application until they became familiar with the use of the application; all participants finished their practice within 15 min. Tests were performed in a soundproof room in the presence of either white noise or babble noise (40 and 60 dB SPL, measured at the subject position) generated using two loud speakers. For statistical validation of the application, forty-two ears (from twenty-one subjects) were tested with PTA and the smartphone.

**Figure 6.**
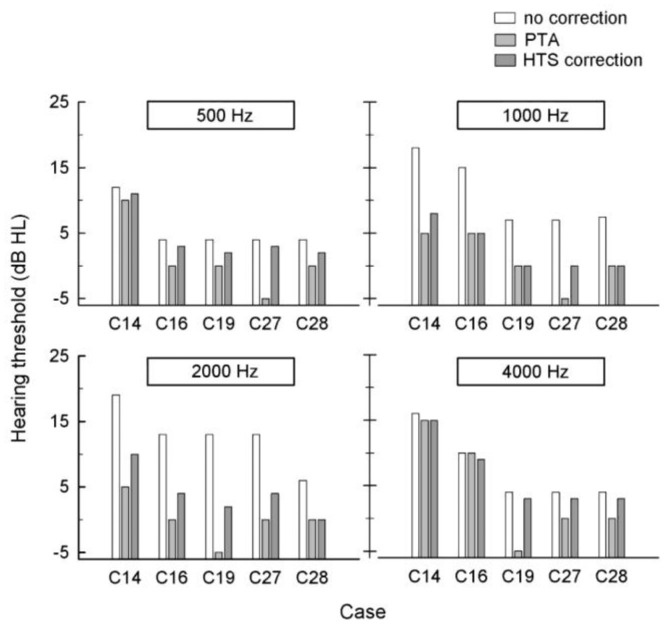
Examples of the performance evaluation of the 500, 1000, 2000, and 4000 Hz tone tests in the presence of 60 dB SPL white noise. White, grey, and dark-grey bars represent the hearing level measured by a smartphone, pure tone audiometry, and a smartphone with the HTS correction algorithm, respectively.

Representative examples are shown in Figure 6. Hearing using the smartphone was tested in the presence of 60 dB SPL babble noise. Each bar shows the results of the hearing threshold obtained from the smartphone test without HTS correction (white bar), PTA (gray bar), and the smartphone test with HTS (dark gray bar) for four tones of 500, 1000, 2000, and 4000 Hz at each panel. The plots indicate that the presence of noise results in an increased hearing threshold. Furthermore, the hearing thresholds without HTS correction are higher than those of PTA and with HTS correction. For example, the hearing threshold by PTA is 0 dB HL for the 1000 Hz tone test for subject test C28, whereas that by a smartphone without HTS correction is 8 dB HL. However, after HTS correction, the hearing threshold by a smartphone is 0 dB HL, which is identical to that of PTA. Although the error in the threshold of each individual varies, the overall data trend shows that a more accurate hearing threshold can be provided by HTS correction.

**Figure 7.**
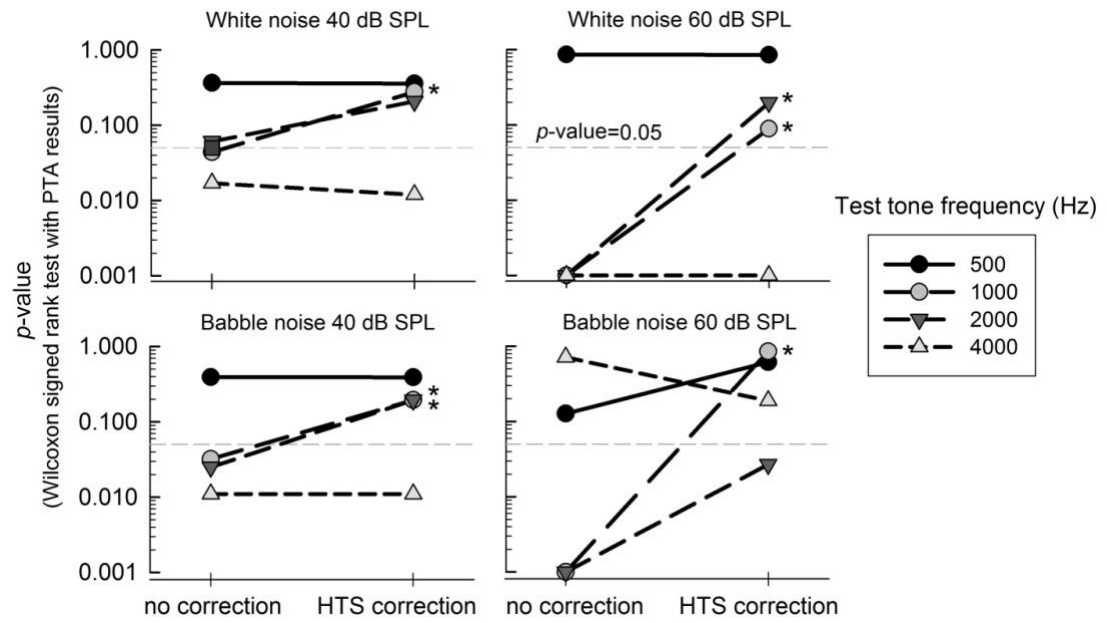
Effect of HTS correction on the similarity with PTA thresholds (n = 42). The environmental noise was white and babble noise at 40 and 60 dB SPL. Each symbol indicates the frequencies of each test tone. See the text for the description of “*”.

A Wilcoxon signed rank test between the PTA results and the smartphone test was performed to validate the smartphone hearing test. As we assumed that the threshold measured by PTA is the reference data, the *p*-value of the paired test revealed how similar the threshold of the smartphone hearing test is with the PTA threshold. If the *p*-value is less than 0.05, there is a statistically significant difference, *i.e.*, the threshold of the smartphone hearing test is not correct. Thus, we expected the *p*-value to increase after HTS correction. Figure 7 shows the effect of HTS correction on the *p*-value. In each panel, environmental noise was set to 40-dB-SPL white noise, 60-dB-SPL white noise, 40-dB-SPL babble noise, and 60-dB-SPL babble noise. The *p*-values between the PTA data and the threshold of the smartphone hearing test with/without HTS correction were calculated. The results show an improvement (increasing *p*-value) at 1000- and 2000-Hz after HTS correction. The *p*-value threshold to categorize the significant difference between two groups was set to 0.05. We represented the effectiveness of HTS correction using the “*” symbol in each panel if the *p*-value changes from a “different threshold” of no correction to a “similar threshold” of HTS correction. There was a significant improvement at 1000 and 2000 Hz for both the white- and babble-noise cases. For example, the *p*-value for 1000 Hz for 60-dB-SPL babble noise changed from 0.001 without HTS correction to 0.850 with HTS correction. This change indicates that the HTS correction improved the similarity of the two thresholds between PTA and the application test. As seen in Figure 7, no improvement was observed for three cases at 4000 Hz; however, there were no cases that exhibited deterioration, *i.e.*, the *p*-value changes from a “similar threshold” of no correction to a “different threshold” of HTS correction.

### 3.4. Hearing Test in a Real-World Environment

**Figure 8.**
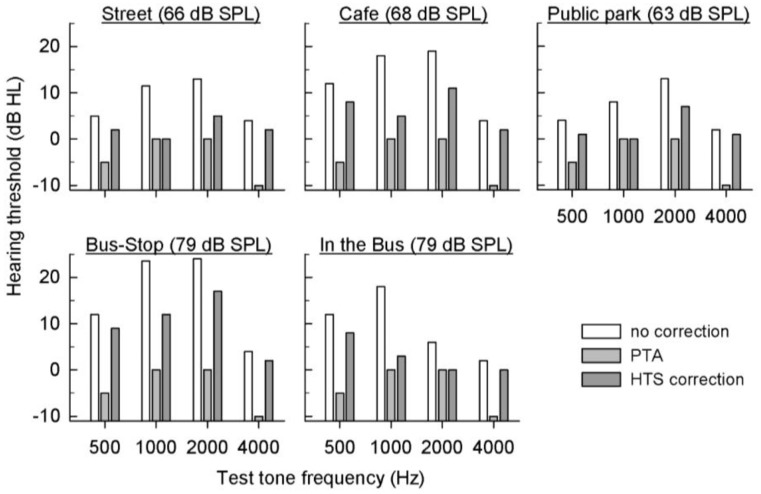
Hearing thresholds of the 500, 1000, 2000, and 4000 Hz tone tests outside the clinic.

The hearing thresholds were measured in a clinic by constructing a noisy environment using a loudspeaker. For a preliminary test of the compatibility of the application in real-world environments, one subject tested their hearing in various environments using the application outside the clinic of on the street; at a cafe, bus-stop, and public park; and inside of a bus. Figure 8 shows the hearing thresholds obtained from the smartphone test without HTS correction (white bar), PTA (gray bar), and the smartphone test with HTS (dark gray bar) for four tones of 500, 1000, 2000, and 4000 Hz. The average noise levels on the street; at the cafe, bus-stop, and public park; and inside of the bus were 66, 68, 79, 63, and 79 dB SPL, respectively. In most cases, the threshold with HTS correction is comparably similar to the PTA threshold.

### 3.5. Evaluation of User Convenience

**Figure 9.**
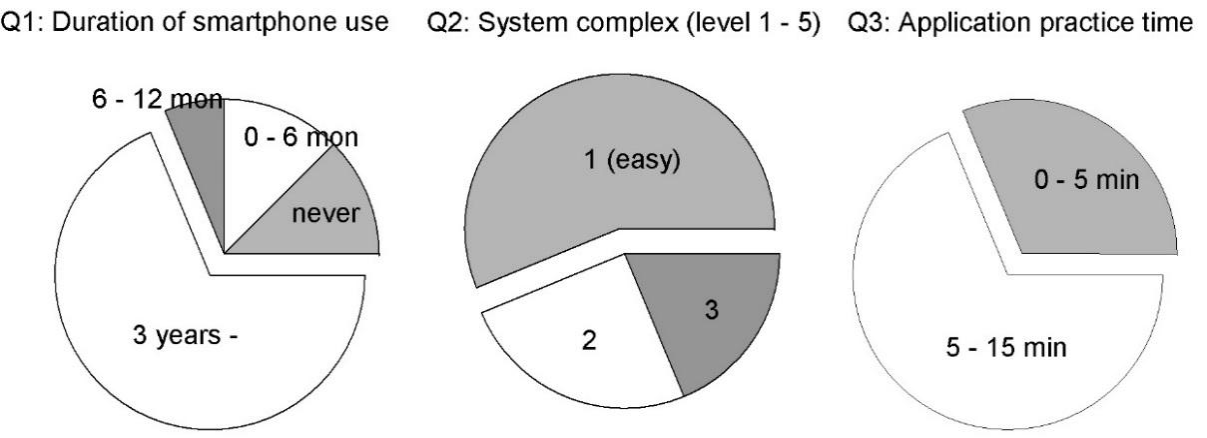
Convenience of the hearing test application (n = 16).

As the advantage of the application is easy accessibility without visiting a clinic, user convenience is an important consideration. A questionnaire-based study was performed to explore the complexity of the application and procedure. Figure 9 summarizes the participants' characteristics and the responses to the questions. The simple questionnaire was administered to sixteen participants. 81% of the people surveyed thought that the application was very easy (or easy) to use. All participants required a practice time of less than 15 min before the hearing test. Thus, it would be helpful to inform potential users of “15 min of practice time prior to the hearing test” as a guide.

## 4. Discussion and Conclusions

### 4.1. Summary

This study demonstrates the development of a hearing-test application and the accessibility in real-life area such as a home, restaurant, or quiet environment. The most innovative idea was the use of the built-in microphone of a smartphone to measure the noise level of the testing environment. The built-in microphone was used to capture environmental sounds. After a pure-tone hearing test was completed, the application reported the hearing thresholds for four frequencies and automatically corrected the HTS on the basis of the level of environmental noise. The preliminary evaluation showed that smartphone-based hearing screening could properly estimate the hearing threshold even in a noisy environment. Typically, HTS correction resulted in a significant improvement at 1000 and 2000 Hz in the hearing test. During the development of the application, we have considered that self-screening should be easily accessible and simple to use. The questionnaire results show that most participants thought that the application was easy to use and needed less than 15 min of practice prior to its use.

### 4.2. Future Directions

More research on this topic needs to be undertaken before distributing the application for public use. In this study, we calibrated the microphone and earphone of a specific smartphone model (Galaxy, Samsung, Suwon, Korea) and an obvious concern is that the developed application may be valid only for this model. However, when we calibrated other smartphones as a preliminary step, the results showed that the calibration tables were not significantly different from those of the smartphone used in this study, but systematic calibrations for the microphone and earphone are essential to increase the accuracy in order to use the application on other models.

In this application, the user was forced to select the type of environmental noise, either white noise or babble noise. Even though the application explains the selection of the noise type, it might be difficult for the user to choose the correct noise type. The user can simply choose babble noise when he/she uses the application at a restaurant, coffee shop, classroom, or indoor area; otherwise, the user can choose white noise. An improvement in the application would be achieved by using more environmental-noise categories. Furthermore, we are developing a noise-recognition algorithm that could automatically categorize the noise type so that the user does not need to choose the noise type. Although the application measured the significantly reliable hearing thresholds at 500, 1000, and 2000 Hz in a noisy environment, there was still a lack of reliable hearing thresholds for the test carried out at 4000 Hz. PTA used a headset to provide the tone sound, whereas the smartphone test used an earphone packed within the smartphone package. Thus, the effects of the concha and external auditory canal, which has a resonant frequency of approximately 2500–5000 Hz [14], will likely produce a difference in thresholds. We expect that a more complex analysis of the resonant effect will increase the accuracy of the threshold at 4000 Hz.

During the validation procedure, eighteen normal-hearing and three mild-hearing-loss subjects participated. Owing to the relatively small number of subjects with hearing loss, there were still limitations for validating the performance of the hearing-loss group. Thus, further data collection using subjects with varying degrees of hearing loss as well as normal hearing is required in future research. More real-world tests are also needed to explore the statistical significance of the real-world suitability of the application.

### 4.3. Clinical Relevance

More than 360 million people around the world suffer hearing loss, and many people are exposed to noisy environments each day. As most people do not have easy access to a clinic, it is difficult to recognize the gradual loss in hearing [15]. Thus, a self-testing application is useful to understand hearing loss and provides advice on the necessity for visiting a clinic for professional pure-tone audiometry. Recently, hearing loss has become a societal issue due to the frequent usage of portable music devices and the aging society [16,17]. Hearing loss may result in decreased physical function, mental health, and cognitive function [18,19]. Hearing impairment may also cause limitations in physical activities such as postural balance and driving performance [20,21]. Early detection and treatment can reduce the negative consequences of hearing loss. We expect that a ubiquitous hearing test using a smart device can provide early identification of hearing loss. Then, the negative consequences of hearing loss may be reduced by proper treatment with a hearing aid, a cochlear implant, surgical correction, or medical management [18].

## Figures and Tables

**Table 1.** Earphone 1000 Hz-output Calibration Table.

**Output** **Smartphone Unit**	**Recording Level**	

	**dB SPL**	**dB HL**
1	13.4	6.9
2	14.3	7.8
3	21.4	14.9
4	27.4	20.9
5	32.4	25.9
6	35.5	29.0
7	38.4	31.9
8	40.5	34.0
9	42.4	35.9
10	44.4	37.9
11	46.4	39.9
12	48.4	41.9
13	50.5	44.0
14	53.4	46.9
15	56.4	49.9

**Table 2.** Microphone Calibration Table.

Babble noise	**0–40 dB SPL**	**40–70 dB SPL**

	$\begin{array}{c} \text{f}={\text{y}}_{0}+\text{ax}+\text{b}x^{2}=cx^{3}\\ \left(\begin{array}{cc} {\text{y}}_{0}=-176.56, & \text{a}=3388.97\\ \text{b}=-19628.85, & \text{c}=39152.25 \end{array}\right) \end{array}$	$\begin{array}{c} \text{f}={\text{y}}_{0}+a\text{ln}\left|x\right|+b{\left(\ln\left|x\right|\right)}^{2}+c{\left(\ln\left|x\right|\right)}^{3}\\ \left(\begin{array}{cc} y_{0}=51.3, & \text{a}=8.61\\ \text{b}=0.86, & \text{c}=-0.27 \end{array}\right) \end{array}$

White noise	**0–50 dB SPL**	**50–80 dB SPL**

	$\begin{array}{c} \text{f}=\text{D}+\left(A-D\right)/\left(1+10^{\left(\left(\left(x-\log C\right)B\right)\right)}\right)\\ \left(\begin{array}{cc} A=50.55, & B=-18.06\\ \log C=0.155, & D=16.00 \end{array}\right) \end{array}$	$\begin{array}{c} \text{f}=y_{0}+a\ln\left|x\right|+b{\left(\ln\left|x\right|\right)}^{2}+c{\left(\ln\left|x\right|\right)}^{3}\\ \left(\begin{array}{cc} y_{0}=63.63, & \text{a}=8.33\\ \text{b}=-0.76, & \text{c}=0.09 \end{array}\right) \end{array}$

## References

[1] Lin F.R., Yaffe K., Xia J., Xue Q.L., Harris T.B., Purchase-Helzner E., Satterfield S., Ayonayon H.N., Ferrucci L., Simonsick E.M. (2013). Hearing loss and cognitive decline in older adults. JAMA Intern. Med..

[2] Davis A., Smith P. (2013). Adult hearing screening: Health policy issues—What happens next?. Am. J. Audiol..

[3] Ramya C.S., Karthiyanee K., Vinutha S. (2011). Effect of mobile phone usage on hearing threshold: A pilot study. Indian J. Otol..

[4] Vogel I., Brug J., Hosli E.J., van der Ploeg C.P., Raat H. (2008). Mp3 players and hearing loss: Adolescents' perceptions of loud music and hearing conservation. J. Pediatr..

[5] Watson C.S., Kidd G.R., Miller J.D., Smits C., Humes L.E. (2012). Telephone screening tests for functionally impaired hearing: Current use in seven countries and development of a us version. J. Am. Acad. Audiol..

[6] Boulos M.N., Wheeler S., Tavares C., Jones R. (2011). How smartphones are changing the face of mobile and participatory healthcare: An overview, with example from ecaalyx. Biomed. Eng. Online.

[7] Mena L.J., Felix V.G., Ostos R., Gonzalez J.A., Cervantes A., Ochoa A., Ruiz C., Ramos R., Maestre G.E. (2013). Mobile personal health system for ambulatory blood pressure monitoring. Comput. Math. Methods Med..

[8] Petersen C.L., Chen T.P., Ansermino J.M., Dumont G.A. (2013). Design and evaluation of a low-cost smartphone pulse oximeter. Sensors.

[9] Macias E., Suarez A., Lloret J. (2013). Mobile sensing systems. Sensors.

[10] Hii P.C., Chung W.Y. (2011). A comprehensive ubiquitous healthcare solution on an android mobile device. Sensors.

[11] Handzel O., Ben-Ari O., Damian D., Priel M.M., Cohen J., Himmelfarb M. (2013). Smartphone-based hearing test as an aid in the initial evaluation of unilateral sudden sensorineural hearing loss. Audiol. Neurootol..

[12] Kam A.C., Sung J.K., Lee T., Wong T.K., van Hasselt A. (2012). Clinical evaluation of a computerized self-administered hearing test. Int. J. Audiol..

[13] Agrawal Y., Platz E.A., Niparko J.K. (2008). Prevalence of hearing loss and differences by demographic characteristics among us adults: Data from the national health and nutrition examination survey, 1999–2004. Arch. Intern. Med..

[14] Yost A.W. (2006). Fundamentals of Hearing.

[15] Margolis R.H., Morgan D.E. (2008). Automated pure-tone audiometry: An analysis of capacity, need, and benefit. Am. J. Audiol..

[16] Catalano P.J., Levin S.M. (1985). Noise-induced hearing loss and portable radios with headphones. Int. J. Pediatr. Otorhinolaryngol..

[17] Sulaiman A.H., Seluakumaran K., Husain R. (2013). Hearing risk associated with the usage of personal listening devices among urban high school students in malaysia. Public Health.

[18] Strawbridge W.J., Wallhagen M.I., Shema S.J., Kaplan G.A. (2000). Negative consequences of hearing impairment in old age: A longitudinal analysis. Gerontologist.

[19] Uhlmann R.F., Larson E.B., Rees T.S., Koepsell T.D., Duckert L.G. (1989). Relationship of hearing impairment to dementia and cognitive dysfunction in older adults. JAMA.

[20] Hickson L., Wood J., Chaparro A., Lacherez P., Marszalek R. (2010). Hearing impairment affects older people's ability to drive in the presence of distracters. J. Am. Geriatr. Soc..

[21] Viljanen A., Kaprio J., Pyykko I., Sorri M., Koskenvuo M., Rantanen T. (2009). Hearing acuity as a predictor of walking difficulties in older women. J. Am. Geriatr. Soc..

